# Barriers to sexual and reproductive health access among migrant women: a cross-sectional study in five regions in Morocco

**DOI:** 10.1136/bmjph-2025-003820

**Published:** 2026-07-21

**Authors:** Oumnia Bouaddi, Jamila Mendili, Hafida Yartaoui, Kenza Hassouni, Sanaa Belabbes, Asmae Khattabi, Hanane Rassimi, Saad Zbiri, Abdelilah El Marnissi, Abdelhakim Yahyane, Radouane Belaouali, Mohamed Khalis

**Affiliations:** 1 Department of Public Health and Clinical Research, Mohammed VI Center for Research and Innovation, Rabat, Morocco; 2 Mohammed VI International School of Public Health, Mohammed VI University of Sciences and Health, Casablanca, Morocco; 3 Ministry of Health and Social Protection, Rabat, Morocco; 4 Higher Institute of Nursing Professions and Health Techniques, Rabat, Morocco

**Keywords:** Public Health, Sexual Health, Health Services Accessibility

## Abstract

**Introduction:**

Morocco, a transit and destination country, has implemented policies granting free primary care and community outreach to improve sexual and reproductive health (SRH) services for migrant women. Despite this, studies suggest that SRH services remain underutilised. This study assessed barriers to SRH services and migrant-supported strategies to improve access.

**Methods:**

We conducted a cross-sectional study in July 2024 in five Moroccan regions with high migrant concentrations. Migrant women of reproductive age were recruited via respondent-driven sampling with local non-governmental organizations (NGOs). A structured questionnaire assessed sociodemographic characteristics, migration profile, SRH barriers and preferred strategies. Bivariate and multivariable logistic regression identified sociodemographic predictors of experiencing barriers.

**Results:**

Overall, 1213 migrant women participated (mean age 29.5, SD 7.7). 80.2% were from Sub-Saharan Africa and 14.0% reported experiencing barriers to SRH services. Undocumented migrants had significantly higher odds of reporting barriers than documented migrants (adjusted OR: 2.21; 95% CI 1.05 to 5.24). Among those reporting barriers (N=170), the most prevalent were absence of health insurance (89.4%), cost of consultations (88.2%), lack of information about services (82.4%) and transport difficulties (68.8%). Cultural and provider-related barriers were rarely reported. Affordability-related interventions were most strongly endorsed: free SRH services (mean 4.56), health insurance coverage (4.55) and free medicines and contraceptives (4.53).

**Conclusions:**

Barriers to SRH access are driven predominantly by structural factors, including financial hardship, administrative constraints, and information gaps, rather than individual or cultural factors, reflecting a the persistence of the policy-practice gap. Addressing this requires financial protection for undocumented migrants, action on upstream access barriers, and innovative financing leveraging Morocco's ongoing health reforms. This should be paired with migrant-sensitive, co-designed communication to empower migrant women and support service navigation.

**WHAT IS ALREADY KNOWN ON THIS TOPIC**

- Migrant women often face increased sexual and reproductive health (SRH) risks and barriers to accessing services in host countries.
- There is limited evidence on barriers to SRH access and on approaches supported by migrants in relatively new destination countries in the Global South, such as Morocco.

**WHAT THIS STUDY ADDS**

- This survey of over 1000 migrant women across five regions found that SRH access is still constrained by economic barriers, administrative issues and a lack of information about entitlements and pathways to care.

**HOW THIS STUDY MIGHT AFFECT RESEARCH, PRACTICE OR POLICY**

- There is a need to develop financial protection mechanisms and raise awareness about available services and access to care, using appropriate communication strategies to promote service utilisation.

## Introduction

 Over the past two decades, Morocco has shifted from being mainly a transit country to becoming a key destination for thousands of migrants.^1^ According to the International Organization for Migration, Morocco hosted approximately 111 069 migrants by mid-2024 representing 0.3% of the international migrant stock,^2,3^ with the majority originating from French-speaking sub-Saharan Africa.^1^ In addition, by the end of 2024, the country hosted over 18 000 people identified as forcibly displaced and in need of protection, and 11 395 asylum-seekers.^4^ Recognising this demographic shift, the Moroccan government has implemented progressive migrant integration policies, most importantly through large-scale regularisation campaigns between 2013 and 2016 that granted legal status to approximately 50,000 migrants.^5,6^

Morocco has also made significant strides in ensuring healthcare access for its growing migrant population. National policies guarantee all migrants, regardless of status, access to a network of primary and emergency healthcare services,^6^ along with inclusion in vertical disease programmes covering tuberculosis (TB) control, immunisation and sexual and reproductive health (SRH), including maternal neo-natal and child health and HIV and blood-borne hepatitis.^7^ The Ministry of Health and Social Protection in collaboration with other stakeholders has also developed the National Strategic Plan for Immigrant Health, which is the guiding policy document for efforts in the migration and health space.^8^ Financial commitments further reflect the prioritisation of migrant health as recent analyses indicate that approximately 5% of Morocco’s annual primary healthcare budget is allocated specifically to migrant and refugee health.^9^ However, despite these institutional efforts, critical gaps persist in service accessibility and utilisation due to multiple access barriers including language, low cultural competency of health professionals, poor awareness about service entitlement.^6,8^ Undocumented migrants, in particular, face increased barriers especially in the secondary and tertiary care levels, mainly due to the absence of consistent medical coverage and fear of discrimination and being refused care due to their status.^8^

SRH has emerged as a key focus area for the health of migrants and refugees, aligning with Morocco’s broader emphasis on women’s health and the increasing trend of the feminisation of migration globally and at the national level.^10^ This focus is supported by existing—although limited—studies showing that female migrants in Morocco experience poor SRH outcomes and high-risk behaviours. For instance, one study in Eastern Morocco reported HIV infection rates three times higher among sexually active female migrants compared with males, a significantly greater prevalence of STI symptoms as well as high levels of unprotected intercourse with casual or commercial partners.^11^ Likewise, another study in Rabat showed a high prevalence of sexual and gender-based violence among this group.^12^ Recognising the specific SRH needs of female migrants, the Ministry of Health and Social Protection provides a key package of services, including free prenatal care and maternal immunisation in primary health centres.^13^ It also collaborates closely with community-based organisations to deliver comprehensive outreach SRH services, including screening, treatment, voluntary counselling and testing and SRH educational activities.^14,15^ Despite this, some studies show that the engagement of migrants with SRH services remains suggesting that despite the availability and some level of entitlement to services, utilisation remains limited, likely due to persisting informal barriers.

Despite increasing attention to migrant health in Morocco, existing research on SRH is limited. Most studies focus narrowly on the disease burden of sexually transmitted infections (mainly HIV), rely on small sample sizes and lack disaggregated data to explore how sociocultural and administrative factors shape healthcare experiences and challenges. This study seeks to address these gaps by examining a large, geographically diverse migrant population. Specifically, it aims to (1) describe the barriers female migrants face in accessing SRH services, with particular attention to sociocultural and demographic differences and (2) identify the policy and practice interventions that best align with migrant communities’ expressed needs and preferences.

## Methods

### Study design and setting

This is a cross-sectional study conducted in July 2024 in five regions of Morocco: Tanger-Tétouan-Al Hoceïma, Casablanca-Settat, Dakhla-Oued Ed-Dahab, Oriental and Rabat-Salé-Kénitra. These regions were selected based on their high concentration of migrants, as documented in various studies and surveys conducted by national technical institutions, including reports by the Moroccan High Commission for Planning.^1^

### Study population and participant recruitment

The study included adult female migrants of reproductive age (18–49 years and older) who had been residing in one of the study sites for more than 3 months. Migrants were defined broadly as individuals who had crossed an international border for any reason, including both documented and undocumented migrants, refugees and asylum seekers.^16^ In this paper, we use the term migrant as an umbrella term to refer to all of the above-mentioned groups. A respondent-driven sampling (RDS) method was used to recruit participants. This approach was chosen because migrant women, particularly undocumented migrants, constitute a hard-to-reach population and would likely not be adequately reached through conventional sampling methods. RDS is specifically designed for such hidden populations as it leverages existing social networks to reach individuals who would otherwise be inaccessible through standard random sampling approaches. This method was implemented in collaboration with a network of NGOs that provide services to migrants. Four initial participants (‘seeds’) were recruited by the NGOs, and each seed was given coupons to refer additional respondents.

### Data collection

Data were collected using a structured questionnaire administered face-to-face by trained primary healthcare professionals at primary care facilities in the study sites. The data collection was conducted using tablets. Staff received both theoretical and practical training on participant recruitment and on the use of data collection tools. Supervisors were appointed to ensure the quality and reliability of data collection in the field. The questionnaire was developed drawing on existing literature on well-documented barriers to healthcare access among migrants, and on public health interventions and approaches in migrant SRH previously synthesised and published by the research team.^17,18^ It comprised four main sections. The first section focused on sociodemographic characteristics and living conditions, including age, country of origin, length of stay, reason for migration, entry point, income source, education level, income level and administrative status. The second section assessed whether participants experienced any barriers to accessing SRH services. Participants were first asked a binary yes/no question: *Have you experienced barriers to accessing sexual and reproductive health services in Morocco?* Participants who responded ‘yes’ were then asked to rate each specific barrier on a 5-point Likert scale ranging from 0 (‘not a barrier’) to 4 (‘always a barrier’). For the purposes of this analysis, a barrier was considered present when a participant rated it as ‘sometimes’, ‘very often’ or ‘always’ (scores ≥2). As participants could endorse multiple barriers simultaneously, we report the frequency of each individual barrier among those who reported experiencing barriers (N=170). Participants who responded ‘no’ to the binary question were not administered the specific barrier items. Finally, to assess participant preferences for interventions to improve SRH access, each proposed intervention, strategy or approach was rated on a 5-point Likert scale ranging from 1 (‘not at all important’) to 5 (‘very important’). Mean importance scores and SD were calculated for each intervention and ranked in descending order to identify the most strongly supported approaches.

### Data analysis

Data were cleaned and checked for quality and completeness. Descriptive analyses were performed: quantitative variables were summarised using means and SD, while qualitative variables were presented as frequencies and percentages. To examine sociodemographic predictors of experiencing barriers to SRH services, we first conducted a bivariate analysis for each variable against the binary outcome. Binary logistic regression was used to estimate crude ORs with 95% CIs and p values for each predictor. Variables were selected for inclusion in the bivariate analysis based on their completeness (absence of missing values) and relevance to barriers to SRH access as informed by the existing literature on healthcare access among migrant populations. Variables with a p-value below 0.05 in the bivariate analysis were then retained for inclusion in a multivariable logistic regression model. Results are reported as crude ORs and adjusted ORs (aOR) with their corresponding 95% CIs and p values. The statistical significance threshold was set at p<0.05 for all analyses. Missing values were excluded on a per-variable basis and are reported in the table. Barriers were organised using the Levesque Healthcare Access framework, which comprises five accessibility and ability dimensions.^18^ This model has been used extensively in healthcare service accessibility research including migrant, refugee and ethnic minority groups. All data management and analysis were conducted using R. This study is reported in accordance with the Strengthening the Reporting of Observational Studies in Epidemiology (STROBE) checklist.^19^

### Ethical considerations

To preserve participant anonymity, address potential discomfort associated with signing formal documents in the participants’ cultural context, and accommodate varying literacy levels among participants, written consent was not obtained, and verbal consent was instead secured by the interviewer after providing a detailed explanation of the study objectives, potential risks, and benefits and addressing any questions. All data were treated with confidentiality in accordance with data protection laws in Morocco, and access to data was restricted to the research team.

### Patient and public involvement

Patients or the public were not involved in the design, or conduct, or reporting or dissemination plans of our research.

## Results

### Sociodemographic characteristics of participants

A total of 1213 migrant women were included in the study (table 1). The mean age was 29.5 years (SD: 7.7), with 77.1% aged below 35 years. The majority were from Sub-Saharan Africa, mainly West Africa (80.2%), with Ivory Coast alone accounting for 52.8% of the study sample. The average household size was 2.8 people (SD: 2.0), and the mean duration of stay in Morocco was approximately 3 years (35.3 months, SD: 26.9). Most participants were single (59.5%), and over 70% had some level of formal education, while 28.0% reported no formal schooling. In terms of income, 31.5% reported earnings below or up to the minimum wage, and 23.7% had no income. For over half of the participants, the main source of income was salary or independent work (53.6%). Only 5.6% had medical insurance or coverage, and two-thirds (67.1%) were living in shared accommodations. Regarding migration status, 77.0% were undocumented, 15.5% had a regular administrative status and only 4.8% had a refugee status. Economic reasons were the main reported driver of migration (58.2%), followed by transit to Europe (10.8%) and the pursuit of education (10.3%).

**Table 1:** Sociodemographic characteristics of participants (N=1213)

Variable	Distribution N (%)
Age mean (SD)	29.5 (7.72)
Age categories (years)	
18–25	448 (36.9)
26–32	388 (23.1)
>32	377 (31.1)
Country and region of origin	
West Africa (N=973)	
Ivory Coast	641 (52.8)
Guinea Conakry	146 (12.0)
Mali	78 (6.4)
Senegal	77 (6.3)
Nigeria	27 (2.2)
Ghana	2 (0.2)
Liberia	2 (0.2)
Central Africa (n=142)	
Cameroun	41 (3.4)
Central African Republic	31 (2.5)
Congo Brazzaville	20 (1.6)
Gabon	11 (0.9)
Democratic Republic of the Congo (DRC)	39 (3.2)
Middle East (Syria)	26 (2.1)
Other	73 (6.0)
Relationship status (N=1209)	
Single	722 (59.5)
Partnered/married	353 (29.0)
Divorced/separated	106 (8.7)
Widowed	29 (2.4)
Prefer not to say	4 (0.3)
Level of education	
None	340 (28.0)
Primary	274 (22.6)
Secondary	232 (19.1)
High school	177 (14.6)
University	190 (15.7)
Monthly income (N=1200)	
[1550 et 3112 DHs[	505 (41.6)
[3112 et 5000 DHs[	33 (2.7)
[5000 et 10 000 DHs[	3 (0.2)
<1550 DHs	372 (30.7)
No income	287 (23.7)
Source of income	
No source of income	247 (20.4)
Salary	374 (30.8)
Independent/individual work	277 (22.8)
Social services/assistance (associations, NGOs)	22 (1.8)
Financial support from family/friends	91 (7.5)
Financial support from spouse/partner	61 (5.0)
Study scholarship	64 (5.3)
Other	77 (6.3)
Has medical coverage/insurance	68 (5.6)
Accommodation type	
Apartment	261 (21.5)
Room in shared accommodation	814 (67.1)
House	26 (2.1)
Without housing	2 (0.2)
Other	109 (9.0)
Household size (mean SD) (N=1102)	2.81 (1.95)
Time of stay in months (mean (SD))	35.3 (26.9)
Up to 18 months	416 (34.3)
18–36 months	403 (33.2)
>36 months	394 (32.5)
Migration status	
Asylum seeker	32 (2.6)
Undocumented	934 (77.0)
Documented	188 (15.5)
Refugee (recognised by the state and/or by UNHCR)	59 (4.8)
Reason for migration	
Employment or seeking employment	706 (58.2)
Political conflict	63 (5.2)
Transit	131 (10.8)
Pursue studies	125 (10.3)
Family reunification	108 (8.9)
Other	80 (6.6)
Entry point	
Casablanca	881 (72.6)
Rabat	57 (4.7)
Oujda	195 (16.1)
Dakhla	52 (4.3)
Others	28 (2.3)

NGOs, non-governmental organisations; UNHCR, United Nations High Commissioner for Refugees.

### Sociodemographic predictors of experiencing barriers to SRH services

Participants aged 26–32 years comprised the largest proportion of those who experienced barriers (40.0%), while among those who did not experience barriers, the 18–25 age group was the largest (38.4%) (table 2). For education level, the distribution was similar across groups, with most participants reporting some level of education (70.0% among those who experienced barriers vs 72.3% among those who did not). The vast majority of participants who experienced barriers were undocumented (91.8%), compared with 74.6% among those who did not. Participants who experienced barriers reported a shorter average duration of stay in Morocco (29.9 months vs 36.2 months). Finally, employment or job search was the main reason for migration in both groups but was more prevalent among those who experienced barriers (79.4% vs 54.7%).

**Table 2:** Bivariate and multivariable analysis: crude and adjusted ORs for sociodemographic predictors of experiencing barriers to SRH services (N=1213)

Variable	Has not experienced barriers (N=1043)	Has experienced barriers (N=170)	Crude OR (95% CI)	P value	aOR (95% CI)	P value
Age (years±SD)	29.4±7.8	30.1±7.2				
Age categories						
18–25 (ref)	400 (38.4)	48 (28.2)	–	–	–	–
26–32	320 (30.7)	68 (40.0)	**1.77 (1.19 to 2.65)**	**0.005**	1.48 (0.98 to 2.26)	0.064
>32	323 (31.0)	54 (31.8)	1.39 (0.92 to 2.12)	0.118	1.40 (0.89 to 2.19)	0.142
Level of education						
Some (ref)	754 (72.3)	119 (70.0)	–	–	–	–
None	289 (27.7)	51 (30.0)	1.12 (0.78 to 1.59)	0.537	—	—
Accommodation type						
Room or shared housing (ref)	687 (65.9)	127 (74.7)	–	–	–	–
Other	356 (34.1)	43 (25.3)	**0.65 (0.45 to 0.94)**	**0.024**	0.83 (0.56 to 1.22)	0.352
Migration status						
Documented (ref)	180 (17.3)	8 (4.7)	–	–	–	–
Refugee/asylum seeker	85 (8.1)	6 (3.5)	1.59 (0.51 to 4.71)	0.405	1.42 (0.45 to 4.29)	0.533
Undocumented	778 (74.6)	156 (91.8)	**4.51 (2.32 to 10.20)**	**<0.001**	**2.21 (1.05 to 5.24)**	**0.050**
Time of stay in months (SD)	36.2 (27.4)	29.9 (23.2)				
Region of origin			–	–	–	–
West Africa (ref)	818 (78.4)	154 (90.6)	**0.36 (0.17 to 0.68)**	**0.004**	0.66 (0.30 to 1.30)	0.257
Central Africa	133 (12.8)	9 (5.3)	**0.40 (0.17 to 0.83)**	**0.024**	0.77 (0.31 to 1.68)	0.545
Other	92 (8.8)	7 (4.1)	**0.99 (0.98 to 1.00)**	**0.005**	0.99 (0.99 to 1.00)	0.094
Reason for migration						
Employment/job search (ref)	571 (54.7)	135 (79.4)	–	–	–	–
Transit to another country	119 (11.4)	12 (7.1)	**0.43 (0.22 to 0.77)**	**0.007**	**0.48 (0.24 to 0.87)**	**0.023**
Other	353 (33.8)	23 (13.5)	**0.28 (0.17 to 0.43)**	**<0.001**	**0.46 (0.27 to 0.77)**	**0.004**

Bold values indicate statistically significant associations (p<0.05). Education level, civil status and income level were not included in the multivariable model as they were not significant in the bivariate analysis (indicated by –).

aOR, adjusted OR; OR, crude OR; Ref, reference category; SRH, sexual and reproductive health.

Table 2 also presents the results of the bivariate and multivariable logistic regression analyses. In the bivariate analysis, age category, accommodation type, migration status, region of origin, time of stay and reason for migration were significantly associated with experiencing barriers. Education level was not significant in the bivariate analysis and was not included in the multivariable model. After adjustment, two variables remained independently and significantly associated with experiencing barriers. Undocumented migrants had significantly higher odds of experiencing barriers compared with documented migrants (aOR: 2.21; 95% CI 1.05 to 5.24; p=0.050). Compared with those who migrated for employment, migrants in transit to another country (aOR: 0.48; 95% CI 0.24 to 0.87; p=0.023) and those with other reasons for migration (aOR: 0.46; 95% CI 0.27 to 0.77; p=0.004) had significantly lower odds of experiencing barriers. Accommodation type, region of origin, time of stay and age category were significant in the bivariate analysis but did not remain significant after adjustment.

### Description of specific barriers to SRH experienced by participants

Table 3 describes the challenges experienced in accessing SRH services among participants. On average, participants endorsed 10.0 barriers simultaneously (SD: 3.5; range: 1–18), indicating the co-occurrence of multiple barriers. At the approachability level, 60.6% reported fear of discrimination and judgement as a barrier, and 57.6% did not know where or whom to contact for SRH services. Previous negative experiences were reported by 37.1%, while lack of trust in health professionals (28.8%) and in quality of care (24.7%) were less commonly reported. At the acceptability level, cultural beliefs (17.1%) and partner’s influence (9.4%) were infrequently reported as barriers. In relation to availability and accommodation, transport difficulties (68.8%), administrative and legal difficulties (68.2%) and lack of family support (67.1%) were the most commonly reported barriers in this dimension, followed by irregular migration status (61.8%), waiting times (57.1%), distance to services (55.9%) and service opening hours (43.5%). The affordability dimension showed the highest burden, with absence of health insurance reported by 89.4% and cost of consultations by 88.2% of those experiencing barriers. For appropriateness and ability to engage, 82.4% reported a lack of information about health services as a barrier, and 62.4% indicated language as a major barrier. The presence of male health staff was rarely reported as a barrier (18.8%).

**Table 3:** Barriers experienced by sub-Saharan African migrant women (N=170)

Levesque dimension	Barrier	Barrier present N (%)	Barrier not present N (%)
Approachability/ability to perceive	Previous negative experience	63 (37.1)	107 (62.9)
	Do not know where or whom to contact	98 (57.6)	72 (42.4)
	Lack of trust in health professionals	49 (28.8)	121 (71.2)
	Lack of trust in the quality of care	42 (24.7)	128 (75.3)
	Fear of discrimination and judgement	103 (60.6)	67 (39.4)
Acceptability/ability to seek	Cultural beliefs about SRH	29 (17.1)	141 (82.9)
	Partner’s influence	16 (9.4)	154 (90.6)
Availability and accommodation/ability to reach	Health service opening hours	74 (43.5)	96 (56.5)
	Distance to health services	95 (55.9)	75 (44.1)
	Transport difficulties	117 (68.8)	53 (31.2)
	Waiting time	97 (57.1)	73 (42.9)
	Administrative and legal difficulties	116 (68.2)	54 (31.8)
	Lack of family support	114 (67.1)	56 (32.9)
	Irregular migration status	105 (61.8)	65 (38.2)
Affordability/ability to pay	Absence of health insurance	152 (89.4)	18 (10.6)
	Cost of consultations/health services	150 (88.2)	20 (11.8)
Appropriateness/ability to engage	Lack of information about health services	140 (82.4)	30 (17.6)
	Language barrier	106 (62.4)	64 (37.6)
	Male health staff	32 (18.8)	138 (81.2)

Analyses restricted to participants who reported experiencing barriers to SRH services (N=170). A barrier was considered present when rated as ‘sometimes’, ‘very often’ or ‘always’ on a five-point Likert scale.

SRH, sexual and reproductive health.

### Strategies and approaches to promote access to SRH services

Table 4 presents the mean importance scores assigned by participants to each proposed intervention, ranked in descending order. The items before scoring have been added to online supplemental table S1. Financial interventions were most strongly supported, with free SRH services (4.56±0.57), health insurance coverage for migrants (4.55±0.57) and free medicines and contraceptive products (4.53±0.58) receiving the highest scores. Facilitating access for migrants in irregular situations ranked fourth (4.34±0.68). Community-based and informational approaches were also highly valued, including community campaigns for early disease diagnosis (4.26±0.75), information and awareness campaigns on SRH (4.22±0.78) and involving migrant community health workers (4.22±0.72). Facilitating transportation (4.17±0.76), diversifying communication languages (4.14±0.89) and training health personnel (4.06±0.92) were also well supported. Reducing waiting times received moderate support (3.88±1.09). The availability of female health personnel was moderately valued (3.42±1.42), while the availability of male health personnel received the lowest mean importance score (2.66±1.35).

**Table 4:** Preferences for interventions and approaches to promote access to SRH services among sub-Saharan African migrant women (N=1213)

Rank	Intervention	Mean (SD)
1	Free SRH services	4.56 (0.57)
2	Health insurance coverage for migrants	4.55 (0.57)
3	Free medicines and contraceptive products	4.53 (0.58)
4	Facilitating access for migrants in irregular situations	4.34 (0.68)
5	Community campaigns for early disease diagnosis and STIs	4.26 (0.75)
6	Information and awareness campaigns on SRH	4.22 (0.78)
7	Involving migrant community health workers	4.22 (0.72)
8	Facilitating transportation to health facilities	4.17 (0.76)
9	Diversifying communication languages	4.14 (0.89)
10	Training health personnel on communication, including SRH	4.06 (0.92)
11	Reducing waiting times	3.88 (1.09)
12	Availability of female health personnel	3.42 (1.42)
13	Availability of male health personnel	2.66 (1.35)

Scores based on a five-point Likert scale ranging from 1 (‘not at all important’) to 5 (‘very important’). Interventions ranked by mean importance score in descending order.

SRH, sexual and reproductive health; STI, Sexually-transmitted infections.

## Discussion

This cross-sectional study among female migrants in Morocco, mainly from sub-Saharan Africa, found that 14% of participants experienced barriers to accessing SRH services. Most participants were young, single and undocumented, had migrated for employment purposes, lacked health insurance and resided in shared accommodation. In the multivariable analysis, undocumented migrants had significantly higher odds of reporting barriers compared with their documented counterparts (aOR: 2.21; 95% CI 1.05 to 5.24), and migrants who had come for employment purposes were more likely to report barriers than those in transit or with other reasons for migration. The barrier profile was dominated by structural challenges: under the Levesque framework’s affordability and availability dimensions, financial and administrative barriers were the most prevalent. Informational and language barriers were also common, pointing to gaps in the approachability and appropriateness dimensions of access. Conversely, barriers in the acceptability dimension, such as cultural concerns and the gender of health staff, were less reported. The intervention preferences expressed by participants closely mirrored this barrier profile, with financial and administrative solutions receiving the strongest support.

We found that undocumented migrant women were two times as likely to experience challenges in accessing SRH services, and facilitating access to services for undocumented migrants received strong support from participants. Global systematic reviews on access to SRH services for migrant populations generally find that reported barriers are often informational,^20–22^ stemming from uncertainty about entitlements created by unclear legal provisions,^22^ even in contexts where legal frameworks granting entitlement to care exist. In Morocco, migrants are granted access for free to primary care services and emergency care and are entitled to access to hospitals under the national hospital law, which has a non-discrimination policy.^23^ Thus, these services are available to this population. However, a literature review in other contexts highlights the role of legal barriers and a tendency among migrants to overuse emergency care rather than primary healthcare services.^24^ This raises important questions about the extent to which migrant populations are informed about their entitlements and the organisation of the healthcare system, an issue also reflected in studies showing suboptimal utilisation of SRH services. For example, a study among sub-Saharan African women in Rabat found that among participants who were pregnant at the time of the study, 44% were not using prenatal care, despite it being offered for free in primary health centres.^12^ Similarly, a national survey of 1700 migrants in Morocco found that less than half were aware of free access to pregnancy care in primary care centres, and only 20% reported using contraception.^25^ A more recent study also reported a higher HIV prevalence (1.7%) among migrants compared with the host population, despite migrants being formally included in Morocco’s national HIV/AIDS and TB programmes.^26^ This underutilisation may be explained by the informational gaps on services reported in this study and highlights the need for interventions specifically aimed at empowering migrant women to seek services and successfully navigate SRH care pathways, both independently and by intensifying the existing peer accompaniment model successfully used by community-based organisations operating in the SRH and women’s health space in Morocco. The finding that migrants who had come for employment purposes were more likely to report barriers than others likely reflect the fragmentation of available support by migration status, as refugees and asylum seekers who were forcibly displaced may benefit from more streamlined access to services through international agencies such as United Nations High Commissioner for Refugees (UNHCR), while economic migrants who are predominantly undocumented, are less likely to be reached by such structured support.

An interesting finding in this study is that the presence of male health staff was not perceived as a barrier; however, the presence of female health staff was slightly more supported than that of male health staff. This contrasts with the existing literature. For example, studies in Switzerland among migrants found that Chinese migrants had strong preferences for female obstetricians and for obstetricians of Asian origin.^27^ Similarly, a literature review in Australia reported that women from migrant and refugee backgrounds expressed a strong preference for female health workers, often due to cultural beliefs about appropriate interactions between men and women.^28^ Studies show that migrant women attended by male health workers often found the experience uncomfortable; vaginal examinations performed by male doctors were cited as particularly distressing.^29,30^ Furthermore, studies show that some migrant women may feel uncomfortable speaking openly with male doctors, which exacerbates existing language difficulties.^31^ A recent ethnographic study in Canada, which involved interviews with physicians, found that although physicians were sympathetic to immigrant women’s requests for female obstetricians, they placed greater emphasis on maintaining gender equity in the medical profession and in wider society and were often reluctant to accommodate requests based on the gender of the healthcare provider.^32^ Overall, research on provider gender preferences in SRH care is mixed.^32–34^ However, some studies suggest that female providers may be preferred for intimate care and empathy, whereas male physicians may be valued for surgery, professionalism and perceived superior technical competence.^34^ This indicates a strong interaction between gender norms and healthcare delivery and highlights the need for health professional training that promotes cultural sensitivity, technical competence and relational skills.

Another key finding is that the cost of care and lack of medical insurance were major challenges to SRH care access, suggesting that out-of-pocket spending may be an important issue. Although primary and emergency care are offered free of charge to migrants in Morocco, the challenges often arise in secondary and tertiary care, where childbirth, specialist consultations, and other services must be paid for, and in the absence of health insurance, this may lead to incurring financial hardship. While vulnerable citizens have access to universal health insurance, no such schemes currently exist for undocumented migrants. A recent analysis assessing Moroccan government spending on migrant health found that approximately 5% of the total annual primary healthcare budget was allocated for migrants and refugees, amounting to 141 652.66 US dollars (USD). For secondary-level care, the costs were between 0.37% and 3% of total hospital costs for two hospitals, and 78 193.53 USD for a teaching hospital.^9^ This level of spending is considerable; however, it is unclear to what extent undocumented migrants benefit from SRH services at the secondary and tertiary levels of care, where service use has an associated cost. Morocco’s ongoing policy development and reforms, aimed at achieving universal health protection in the coming years, present a key opportunity to develop innovative financing mechanisms to increase financial protection for migrants, particularly undocumented migrant women who have been shown to experience high SRH risks and maternal morbidity.^12^

While this study examines barriers and solutions to SRH services in a large sample of migrant women in Morocco and contributes by shedding light on perceived barriers specific to SRH, which has not been done before, it has some limitations. First, data collection was mainly conducted in primary care facilities, which may have skewed the results towards migrants who already have some level of access and integration into healthcare services. Second, this study assessed perceived barriers to SRH services broadly and did not examine barriers specific to individual service types such as contraception, HIV care, maternal health or gender-based violence services, which may each present unique access challenges. Third, English-speaking migrants are under-represented and may experience different access barriers, particularly informational ones, and may also be more socioeconomically vulnerable due to greater difficulty accessing both the formal and informal economy. Fourth, this study does not examine granular access to specific SRH services. While most of the literature has focused on HIV, future research should explore specific barriers (and approaches) to screening services for other sexually transmitted infections (STIs) such as HPV as well as access to quality prenatal, antenatal, and postnatal care. There is also room for research exploring innovative approaches to addressing the identified barriers to SRH services in the Moroccan context through community engagement and the coproduction of interventions with migrant communities and other stakeholders, which have been shown to be effective in improving the reach and relevance of healthcare interventions among underserved populations.^35^

## Conclusions

The accessibility of SRH services to migrant women in Morocco appears to be mainly shaped by financial, economic, and administrative constraints alongside limited information and confusion around rights, entitlements, and care pathways. Addressing these barriers requires implementing financial protection measures, especially for undocumented migrants, to protect against catastrophic financial expenditures, while also tackling the broader upstream factors that affect demand for and the ability to access and engage with services. Morocco’s ongoing healthcare system decentralisation and health financing reforms create a timely opening to design innovative financing solutions tailored to this population. Yet how these initiatives are communicated matters just as much as how they are designed and delivered. Migrant-sensitive communication approaches, therefore, need to be co-developed in partnership with the communities.

## Supplementary material

10.1136/bmjph-2025-003820 online supplemental file 1

## Data Availability

Data are available upon reasonable request.
