# Supplementary material for: Barriers to sexual and reproductive health access among migrant women: a cross-sectional study in five regions in Morocco
Source: BMJ Public Health. 2026 Jul 21;4(3):e003820. doi: 10.1136/bmjph-2025-003820 (PMC13404504; doi:10.1136/bmjph-2025-003820)
Supplement: online supplemental file 1 [file bmjph-4-3-s001.docx]

**Table S1. Interventions to promote access to sexual and reproductive health services supported by sub-Saharan African female migrants (N=1213)**

| Intervention | Distribution N (%) | | | | |
| --- | --- | --- | --- | --- | --- |
|  | **Not at all important** | **Slightly important** | **Neutral/No opinion** | **Important** | **Very important** |
| Providing free sexual and reproductive health services | 3 (0.2) | 4 (0.3) | 15 (1.2) | 482 (39.7) | 709 (58.5) |
| Providing health insurance coverage for migrants | 1 (0.1) | 7 (0.6) | 19 (1.6) | 485 (40.0) | 701 (57.8) |
| Providing free medicines and contraceptive products | 2 (0.2) | 5 (0.4) | 28 (2.3) | 490 (40.4) | 688 (56.7) |
| Facilitating transportation of migrants to health facilities | 10 (0.8) | 49 (4) | 56 (4.6) | 713 (58.8) | 385 (31.7) |
| Providing information and raising awareness among migrant populations about sexual and reproductive health services | 14 (1.2) | 41 (3.4) | 54 (4.5) | 661 (54.5) | 443 (36.5) |
| Conducting community campaigns for early diagnosis of diseases and sexually transmitted infections | 10 (0.8) | 38 (3.1) | 54 (4.5) | 637 (52.5) | 474 (39.1) |
| Involving migrant community health workers in outreach and service delivery | 11 (0.9) | 26 (2.1) | 67 (5.5) | 692 (57.0) | 417 (34.4) |
| Diversifying communication languages to improve accessibility | 33 (2.7) | 55 (4.5) | 47 (3.9) | 654 (53.9) | 424 (35.0) |
| Facilitating access for migrants in irregular situations | 9 (0.7) | 20 (1.6) | 27 (2.2) | 645 (53.2) | 512 (42.2) |
| Training health personnel on communication, including sensitive aspects of sexual and reproductive health | 30 (2.5) | 70 (5.8) | 89 (7.3) | 636 (52.4) | 388 (32.0) |
| Reducing waiting times in health facilities | 54 (4.5) | 132 (10.9) | 81 (6.7) | 582 (48.0) | 364 (30.0) |
| Ensuring availability of female health personnel | 190 (15.7) | 183 (15.1) | 89 (7.3) | 432 (35.6) | 319 (26.3) |
| Ensuring availability of male health personnel | 299 (24.6) | 355 (29.3) | 143 (11.8) | 287 (23.7) | 129 (10.6) |
